# Barcoding of Central European *Cryptops* centipedes reveals large interspecific distances with ghost lineages and new species records from Germany and Austria (Chilopoda, Scolopendromorpha)

**DOI:** 10.3897/zookeys.564.7535

**Published:** 2016-02-16

**Authors:** Thomas Wesener, Karin Voigtländer, Peter Decker, Jan Philip Oeyen, Jörg Spelda

**Affiliations:** 1 Zoologisches Forschungsmuseum Alexander Koenig, Leibniz Institute for Animal Biodiversity, Center for Taxonomy and Evolutionary Research (Section Myriapoda), Adenauerallee 160, 53113 Bonn, Germany; 2 Senckenberg Museum of Natural History Görlitz, Am Museum 1, 02826 Görlitz, Germany; 3 Bavarian State Collection of Zoology, Münchhausenstraße 21, 81247 Munich, Germany

**Keywords:** Barcode, biodiversity, COI, cryptic diversity, introduced species

## Abstract

In order to evaluate the diversity of Central European Myriapoda species in the course of the German Barcode of Life project, 61 cytochrome *c* oxidase I sequences of the genus *Cryptops* Leach, 1815, a centipede genus of the order Scolopendromorpha, were successfully sequenced and analyzed. One sequence of *Scolopendra cingulata* Latreille, 1829 and one of *Theatops erythrocephalus* Koch, 1847 were utilized as outgroups. Instead of the expected three species (*Cryptops parisi* Brolemann, 1920; *Cryptops anomalans* Newport, 1844; *Cryptops hortensis* (Donovan, 1810)), analyzed samples included eight to ten species. Of the eight clearly distinguishable morphospecies of *Cryptops*, five (*Cryptops parisi*; *Cryptops croaticus* Verhoeff, 1931; *Cryptops anomalans*; *Cryptops umbricus* Verhoeff, 1931; *Cryptops hortensis*) could be tentatively determined to species level, while a further three remain undetermined (one each from Germany, Austria and Croatia, and Slovenia). *Cryptops croaticus* is recorded for the first time from Austria. A single specimen (previously suspected as being *Cryptops anomalans*), was redetermined as *Cryptops umbricus* Verhoeff, 1931, a first record for Germany. All analyzed *Cryptops* species are monophyletic and show large genetic distances from one another (p-distances of 13.7–22.2%). Clear barcoding gaps are present in lineages represented by >10 specimens, highlighting the usefulness of the barcoding method for evaluating species diversity in centipedes. German specimens formally assigned to *Cryptops parisi* are divided into three clades differing by 8.4–11.3% from one another; their intra-lineage genetic distance is much lower at 0–1.1%. The three clades are geographically separate, indicating that they might represent distinct species. Aside from *Cryptops parisi*, intraspecific distances of *Cryptops* spp. in Central Europe are low (<3.3%).

## Introduction

The German Barcode of Life project – Myriapoda was started in 2012 with the aim to construct a library of reference sequences from the 200 indigenous Diplopoda and Chilopoda species of Germany (Voigtländer et al. 2011). This project, spearheaded by a study of Bavarian myriapods (Spelda et al. 2011), is still in progress. First results of the “German Myriapod Barcoding Group” were presented by Wesener et al. (2015). With the help of a comprehensive gene database, the taxonomical problems and confusion that exists in many myriapod groups on a species and higher level could be solved in combination with morphological character analyses. Additionally, barcoding could make it possible to determine juvenile and female myriapods; such a determination is often impossible with morphological characters only. Furthermore, in combination with other genetic markers, barcoding might allow analyses of the evolutionary history of species or species groups (e.g. Pilz et al. 2007, Oeyen et al. 2014).

Such a problem of taxonomic confusion applies in particular to the family Cryptopidae of the centipede order Scolopendromorpha. The Cryptopidae show an almost worldwide distribution, as they are present on most continents and many islands (Attems 1930). The family shows their highest diversity in the temperate parts of North and South America, Europe and the Mediterranean region, central and southern Africa, Madagascar, and Australia (Bonato and Zapparoli 2011). Many cryptopid taxa are currently difficult to determine and are in need of revisions. While the phylogeny of the family inside the Scolopendromorpha is still not fully resolved (e.g. Murienne et al. 2010; Vahtera et al. 2013), the monophyly of the diverse and cosmopolitan genus *Cryptops* is currently undisputed (Vahtera et al. 2012).

In Germany and most of Central Europe, the only Scolopendromorpha that occur naturally are two widely distributed species of the genus *Cryptops*: *Cryptops parisi* and *Cryptops hortensis* (Voigtländer et al. 2011). Both species are morphologically distinct and relatively easy to identify, at least in the adult stage. However, in the Austrian Inn-valley, unusual specimens previously assigned to *Cryptops hortensis* have been found (Pichler 1987) which might be different from *Cryptops hortensis*, and in later studies were placed in keys (Lewis 2011) under *Cryptops parisi*.

A third species, *Cryptops anomalans*, is a recent addition to the German fauna (Voigtländer 1988; Fründ 1989; Spelda 2006, Decker and Hannig 2011). Although already mentioned as a possible member of the German fauna by Schubart (1964) this species was most likely introduced from the Mediterranean realm to northern Europe (Eason 1964; Lindner 2005), as it is mainly confined to parks and gardens. Because the species has few records in Germany (Decker et al. 2014), a special effort was undertaken to collect specimens from the limited number of known German populations.

There are only a handful of barcoding and phylogenetic studies applying molecular data of Scolopendromorpha worldwide (Murienne et al. 2010; Simaiakis et al. 2012; Vahtera et al. 2012, 2013; Joshi and Edgecombe 2013; Oeyen et al. 2014; Siriwut et al. 2015). For *Cryptops*, there is only a singular molecular study utilizing barcoding genes and it deals with tropical pacific island species (Murienne et al. 2011). Therefore, this study focusing on Central European/German *Cryptops* is the first of its kind.

Barcoding studies inside the Scolopendromorpha consecutively revealed large interspecific distances (Simaiakis et al. 2012; Joshi and Edgecombe 2013; Oeyen et al. 2014; Siriwut et al. 2015). The only study involving *Cryptops* (Murienne et al. 2011) revealed exceptionally high intra- and interspecific distances, similar to the observations made in other Scolopendromorpha genera (see above), as well as in a recent study on German geophilomorph centipedes (Wesener et al. 2015).

The aim of this study is to see if barcoding of *Cryptops* allows (a) a clear separation of the species found in Germany; (b) enables the detection of potential cryptic lineages in the widespread German species; as well as (c) facilitating the correct identification of morphologically distinct specimens from Central Europe.

## Material and methods

### Specimen collection and preparation

**Figure 1.**
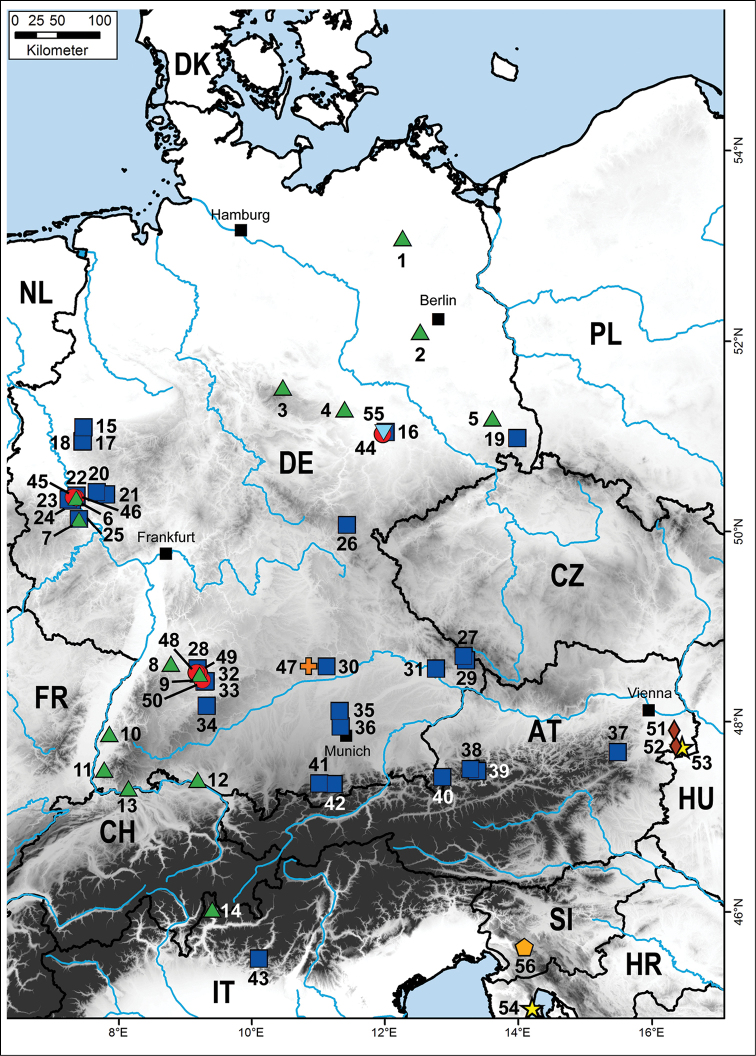
Distribution map of all successfully sequenced Central European specimens of *Cryptops*. Numbers refer to each specimen (see Table 1). Symbols and colours denote species. Blue rectangle = *Cryptops parisi*; red circle = *Cryptops anomalans*; green triangle = *Cryptops hortensis*; brown diamond = *Cryptops croaticus*; orange cross = *Cryptops umbricus*; light blue, orange, and yellow symbols mark undetermined *Cryptops* species.

The focus of the project was *Cryptops* from Germany, which encompass 85% of the here analysed specimens of the genus (Fig. 1). The remaining 15% (11) successfully sequenced specimens of *Cryptops* were collected in adjacent countries. Our sample includes six specimens from Austria, two from Italy, and one each from Croatia, Wales, and Slovenia. One of the Italian specimens is of special importance as it came from the type locality of the subspecies *Cryptops parisi sebini* Verhoeff, 1934. All specimens are stored as vouchers in 95% undenatured ethanol, either at the Museum Koenig, Bonn, Germany (ZFMK), the Senckenberg Museum für Naturkunde, Görlitz (SMNG) or the Bavarian State Collection of Zoology, Munich, ZSM (see Table 1, full specimen information in Suppl. material 1).

**Table 1.** GBOL numbers, GenBank codes, locality data. GBOL number refers to DNA extraction and BOLD registration; L Nr refers to number of Map (Figure 1). SMNG = Senckenberg Museum für Naturkunde, Görlitz, Germany; ZFMK = Zoological Research Museum A. Koenig, Bonn, Germany; ZSM = Zoologische Staatssammlung München, Germany.

L Nr	GBOL	GenBank	Voucher	Species	Locality
	GBOL02755	KU497147	ZSM-ART-JSP130822-001	*Scolopendra cingulata*	Croatia, Istra, Umag
	GBOL02750	KU497149	ZSM-ART-JSP110424-007	*Theatops erythrocephalus*	Croatia, Istra, Brestova
1	ZFMK-TIS-2531556	KM491707	ZFMK-MYR 3450	*Cryptops hortensis*	Germany, Waren (Müritz), Nationalpark Müritz
1	ZFMK-TIS-2531557	KM491678	ZFMK-MYR 3438	*Cryptops hortensis*	Germany, Waren (Müritz), Nationalpark Müritz
2	ZFMK-TIS-1470	KU342047	ZFMK-MYR 3853	*Cryptops hortensis*	Germany, Potsdam, Babelsberg
2	ZFMK-TIS-2507217	KU342045	ZFMK-MYR 3888	*Cryptops hortensis*	Germany, Potsdam, Babelsberg
3	ZFMK-TIS-1543	KM491700	ZFMK-MYR 3684	*Cryptops hortensis*	Germany, Ilsenburg
4	ZFMK-TIS-1528	KM491595	ZFMK-MYR 3679	*Cryptops hortensis*	Germany, Friedeburg (Saale)
4	ZFMK-TIS-2519823	KM491677	ZFMK-MYR 3824	*Cryptops hortensis*	Germany, Friedeburg (Saale)
5	ZFMK-TIS-1289	KU342043	ZFMK-MYR 3551	*Cryptops hortensis*	Germany, Hoyerswerda, Dubringer Moor
6	ZFMK-TIS-15761	KM491615	ZFMK-MYR 1057	*Cryptops hortensis*	Germany, Bonn - Bad Godesberg, Panoramapark
7	ZFMK-TIS-15555	KU342044	ZFMK-MYR 1043	*Cryptops hortensis*	Germany, Niederzissen, Bausenberg
8	GBOL14853	KU497144	ZSM-ART-JSP130930-017	*Cryptops hortensis*	Germany, Enzberg, Kieselbronn
9	GBOL02747	KU497160	ZSM-ART-JSP110312-009	*Cryptops hortensis*	Germany, Zuckerberg SW Stuttgart-Steinhaldenfeld
9	GBOL10885	KU497162	ZSM-ART-JSP110312-009b	*Cryptops hortensis*	Germany, Zuckerberg SW Stuttgart-Steinhaldenfeld
10	GBOL14855	KU497145	ZSM-ART-JSP150118-018	*Cryptops hortensis*	Germany, Kenzingen, Forlenwald
11	GBOL14854	KU497155	ZSM-ART-JSP150117-055	*Cryptops hortensis*	Germany, Badenweiler, Schweighof (Eselsgrabenfelsen),
12	ZFMK-DNA-112780039	KM491565	ZSM-ART-JSP100619-031	*Cryptops hortensis*	Germany, Mainau island, 4 km NNE Konstanz
13	GBOL14858	KU497146	ZSM-ART-JSP150121-039	*Cryptops hortensis*	Germany, Mainau island, 4 km NNE Konstanz
14	ZFMK-DNA-112780041	KU342046	ZSM-ART-JSP110208-005	*Cryptops hortensis*	Italy, Provincia di Sondrio, Chiavenna, Riserva Naturale Marmitte dei Giganti
15	ZFMK-TIS-19439	KM491610	ZFMK-MYR 1948	*Cryptops parisi*	Germany, Bochum, Botanical Garden of the Ruhr-University
16	ZFMK-TIS-1619		ZFMK-TIS-1619	*Cryptops parisi*	Germany, Leipzig-Schönefeld, Partheaue
17	ZFMK-TIS-15786	KM491698	ZFMK-MYR 1082	*Cryptops parisi*	Germany, Schwelm-Erlen, nahe Eingang Erlenhöhle,
18	ZFMK-TIS-15767	KM491624	ZFMK-MYR 1063	*Cryptops parisi*	Germany, Wuppertal, NSG ‚Im Hölken‘
19	ZFMK-TIS-6357	KM491666	ZFMK-MYR 3535	*Cryptops parisi*	Germany, Weißenberg, Gröditzer Skala
20	ZFMK-TIS-2517115	KU342051	ZFMK-MYR 2157	*Cryptops parisi*	Germany, Stromberg (Windeck)
21	ZFMK-TIS-19435	KM491556	ZFMK-MYR 2020	*Cryptops parisi*	Germany, Seelbach bei Hamm (Sieg), Marienthal
21	ZFMK-TIS-19436	KM491664	ZFMK-MYR 2019	*Cryptops parisi*	Germany, Seelbach bei Hamm (Sieg), Marienthal
22	ZFMK-TIS-15462	KM491557	ZFMK-MYR 950	*Cryptops parisi*	Germany, Bonn - Oberkassel, unterhalb Steinbruch,
23	ZFMK-TIS-19593	KM491702	ZFMK-MYR 1545	*Cryptops parisi*	Germany, Bonn - Röttgen, Kottenforst, Naturwaldzelle‚ Oberm Jägerkreuz‘
24	ZFMK-TIS-19592	KM491590	ZFMK-MYR 1544	*Cryptops parisi*	Germany, Wachtberg, Kottenforst bei Pech
25	ZFMK-TIS-15753	KM491588	ZFMK-MYR 1045	*Cryptops parisi*	Germany, Niederzissen, Bausenberg
26	ZFMK-TIS-1561	KU342054	ZFMK-MYR 3697	*Cryptops parisi*	Germany, Lichtenberg, NSG Höllental
27	GBOL14862	KU497148	ZSM-ART-JSP150201-159	*Cryptops parisi*	Germany, Lusen, Winterweg
28	ZFMK-TIS-2520349	KM491592	SMNG VNR016538-3	*Cryptops parisi*	Germany, Ludwigsburg, Salonwald
29	GBOL14843	KU497154	ZSM-ART-JSP130903-006	*Cryptops parisi*	Germany, Felswandergebiet (siev.) 4 km E Neuschoenau, 10 km NE Grafenau
30	GBOL14863	KU497157	ZSM-ART-SSP130614-044	*Cryptops parisi*	Germany, 1 km SE Pfuenz, 7 km ESE Eichstaett
31	GBOL11259	KU497163	ZSM-ART-JSP141004-021	*Cryptops parisi*	Germany, W Unterfrohnstetten, 4 km NNW Hengersberg
32	BCZSMMYR00490	JN266284	ZSM-ART-JSP100508-007	*Cryptops parisi*	Germany, Esslingen-St. Bernhard, Laienweg 33
32	GBOL11266	KU497150	ZSM-ART-JSP130530-002	*Cryptops parisi*	Germany, Esslingen-St. Bernhard, Laienweg 33
33	GBOL14856	KU497152	ZSM-ART-JSP150118-024	*Cryptops parisi*	Germany, Esslinger Burg N Esslingen-Stadtmitte
34	GBOL14859	KU497161	ZSM-ART-JSP150124-038	*Cryptops parisi*	Germany, St. Johann-Fohlenhof, 4 km WSW Bad Urach
35	ZFMK-DNA-112780049	KM491560	ZSM-ART-JSP100516-001	*Cryptops parisi*	Germany, Wendelstein, Ueber der Glonn, 1 km WSW Glonnbercha
36	GBOL02712	KU497164	ZSM-ART-JSP130609-018	*Cryptops parisi*	Germany, Schwarzhoelzl, 2 km NE Karlsfeld
37	ZFMK-TIS-9712	KU342050	ZFMK-MYR 1225	*Cryptops parisi*	Austria, Schneeberg unten
38	BCZSMMYR00493	JN266285	ZSM-ART-JSP100905-017	*Cryptops parisi*	Austria, NW Weinbachbauernhof 1 km NE Strobl, 8 km WNW Bad Ischl
39	GBOL14860	KU497156	ZSM-ART-JSP150124-074	*Cryptops parisi*	Austria, Kaltenbach NNE Ruine Wildenstein, 1 km SW Bad Ischl
40	GBOL14861	KU497141	ZSM-ART-JSP150201-104	*Cryptops parisi*	Germany, W slope of Lercheck, 1 km NW Unterau, 5 km NE Berchtesgaden
41	GBOL02742	KU497140	ZSM-ART-JSP130522-015	*Cryptops parisi*	Germany, SW Grafenaschau, 8 km SW Murnau
42	ZFMK-DNA-112780073	KU342053	ZSM-ART-JSP100510-004	*Cryptops parisi*	Germany, Bad Toelz, Altjoch
43	ZFMK-TIS-2517130	KU342055	ZFMK-MYR 2470	*Cryptops parisi sebini*	Italy, Lombardia, Brescia, Pisogne, Type locality
	GBOL12332	KU497142	ZSM-ART-JSP141214-001	*Cryptops parisi*	UK, Wales, Aberbargoed,
44	ZFMK-TIS-1587	KM491706	ZFMK-MYR 4072	*Cryptops anomalans*	Germany, Leipzig, Pleißemühlgraben
45	ZFMK-TIS-18969	KM491703	ZFMK-MYR 1379	*Cryptops anomalans*	Germany, Bonn, Friesdorf
46	ZFMK-TIS-15751	KM491699	ZFMK-MYR 1047	*Cryptops anomalans*	Germany, Bonn - Bad Godesberg, Panoramapark
46	ZFMK-TIS-15752	KM491639	ZFMK-MYR 1048	*Cryptops anomalans*	Germany, Bonn - Bad Godesberg, Panoramapark
47	BCZSMMYR00489	JN266286	ZSM-ART-JSP100619-017	*Cryptops umbricus*	Germany, Langenaltheimer Haardt 1 km W Solnhofen, 4 km S Pappenheim
48	GBOL02745	KU497151	ZSM-ART-JSP130812-004	*Cryptops anomalans*	Germany, Hummelgraben, Stuttgart-Zuffenhausen
49	GBOL14852	KU497158	ZSM-ART-JSP110624-001	*Cryptops anomalans*	Germany, SW Stuttgart-Muehlhausen
50	GBOL14950	KU497159	ZSM-ART-JSP141105-017	*Cryptops anomalans*	Germany, Ailenberg SE Stuttgart-Obertuerkheim, 1 km WSW Ruedern
51	ZFMK-TIS-2517180	KU342049	ZFMK-MYR 3320	*Cryptops croaticus*	Austria, Leithagebirge, Zeiler Berg
52	ZFMK-TIS-9466	KU342048	ZFMK-MYR 1236	*Cryptops croaticus*	Austria, Leithagebirge I
53	ZFMK-TIS-9755	KM491620	ZFMK-MYR-1185	*Cryptops* sp.	Austria, Burgenland, Rosaliakapelle
54	GBOL14960	KU497153	ZSM-ART-JSP110425-008	*Cryptops* sp.	Croatia, NW Baci and Brestova, 10 km NE Labin
55	ZFMK-TIS-1434	KU342042	ZFMK-MYR 3662	*Cryptops* sp.	Germany, Saxony, Leipzig, Zoo, Gondwanaland
56	GBOL14857	KU497143	ZSM-ART-JSP150118-047	*Cryptops* sp.	Slovenia, Osojca 2 km NW Zagon, 5 km NW Postojna

The specimens were collected by hand and transferred to vials containing 95% undenatured ethanol within days of collection. The vials contain an individual GBOL number with which the specimens can be connected to the accompanying data. After conservation the specimens were either sent to the GBOL facility at the ZFMK or to the corresponding laboratory at the ZSM. Upon arrival, all specimens were photographed (images are or will be uploaded to BOLD, http://www.boldsystems.org/), and a tissue sample was removed for DNA extraction. For this specific GBOL subproject, DNA extraction was attempted for 77 specimens of *Cryptops* as well as one each of *Scolopendra cingulata* and *Theatops erythrocephalus* as outgroups (See Table 1).

Maps were created with ArcGIS 10.

### DNA extraction and sequencing

At the ZFMK, DNA was extracted from the tissue samples using the BioSprint96 magnetic bead extractor by Qiagen (Germany). After the extraction, samples were outsourced for PCR and sequencing (BGI China). For PCR and sequencing, the degenerated primer pair HCOJJ/LCOJJ (Astrin and Stüben 2008) was used, resulting in a success rate of >75% (38 of 49 extracted specimens).

At the ZSM, a single leg was removed from each specimen and sent in 96 well lysis plates to the Canadian Centre for DNA Barcoding (CCDB, Guelph, Canada) for standardized, high-throughput DNA extraction, PCR amplification and bidirectional Sanger sequencing (http://www.ccdb.ca/resources.php). For PCR and sequencing, a primer cocktail (Hebert et al. 2004, see Table 2) was used, resulting in a success rate of >90% (23 from 25 extracted specimens). All voucher information and the DNA barcode sequences, primer pairs and trace files were uploaded to BOLD (http://www.boldsystems.org).

**Table 2.** List of primers used for amplification and sequencing of the 5’ part of the mitochondrial COI gene.

Primer name	Sequence	Publication	Used at
LCO1490	5‘-GGTCAACAAATCATAAAGATATTGG	Folmer et al. 1994	CCDB for ZSM
HCO2198	5‘-TAAACTTCAGGGTGACCAAAAAATCA	Folmer et al. 1994	CCDB for ZSM
LepF1	5‘-ATTCAACCAATCATAAAGATATTGG	Hebert et. al. 2004	CCDB for ZSM
LepR1	5‘-TAAACTTCTGGATGTCCAAAAAATCA	Hebert et. al. 2004	CCDB for ZSM
C_LepFolF	cocktail of LepF1 and LCO1490	www.boldsystems.org/index.php/Public_Primer_PrimerSearch	CCDB for ZSM
C_LepFolR	cocktail of LepR1 and HCO2198	www.boldsystems.org/index.php/Public_Primer_PrimerSearch	CCDB for ZSM
LCO1490-JJ	5‘-CHACWAAYCATAAAGATATYGG	Astrin and Stüben 2008	ZFMK
HCO2198-JJ	5‘-AWACTTCVGGRTGVCCAAARAATCA	Astrin and Stüben 2008	ZFMK

Sequences were obtained for 61 *Cryptops* as well as the two outgroup specimens. The three available sequences of Central European *Cryptops* were added from a previously published dataset (Spelda et al. 2011). Sequence identities were confirmed with BLAST searches (Altschul et al. 1997). All 63 new sequences were deposited in GenBank (see Table 1 for accession numbers). In order to rule-out the accidental amplification of nuclear copies of the mitochondrial COI gene, the whole dataset was translated into amino acids (see Supplemental Material) following the ‘invertebrate’ code in MEGA6 (Tamura et al. 2013); internal stop codons were absent in our dataset. There were a total of 657 positions in the final dataset, gaps were absent.

### Phylogenetic analysis

Sequences were aligned by hand in Bioedit (Hall 1999). The final dataset included 66 nucleotide sequences with 657 positions (63 newly sequenced). Phylogenetic analyses were conducted in MEGA6 (Tamura et al. 2013). A Modeltest, as implemented in MEGA6 (Tamura et al. 2013), was performed to find the best fitting maximum likelihood substitution model. Models with the lowest BIC scores (Bayesian Information Criterion) are considered to describe the best substitution pattern. Included codon positions were 1st+2nd+3rd+Noncoding. Modeltest selected the General Time Reversible model (Nei and Kumar 2000) with gamma distribution and invariant sites as best fitting model (lnL -4725.286624, Invariant 0.505, Gamma 1.65919, R 3.11, Freq A: 0.2844, T: 0.3433, C: 0.2113, G: 0.1606). The tree with the highest log likelihood (-4725.2866) is used here to infer the genetic distances and evolutionary history of the analyzed specimens. Initial tree(s) for the heuristic search were obtained automatically by applying Neighbor-Join and BioNJ algorithms to a matrix of pairwise distances estimated using the Maximum Composite Likelihood (MCL) approach, and then selecting the topology with superior log likelihood value. A discrete Gamma distribution was used to model evolutionary rate differences among sites (5 categories (+G, parameter = 1.6591)). The rate variation model allowed for some sites to be evolutionarily invariable ((+I), 50.5% sites). The bootstrap consensus tree inferred from 1000 replicates (Felsenstein 1985) is taken to represent the evolutionary history of the analyzed taxa. The tree is drawn to scale, with branch lengths measured in the number of substitutions per site.

### Distance analysis

The number of base differences per site between sequences is shown in figures and tables (Fig. 3; Suppl. material 2). The analysis involved 66 nucleotide sequences. Codon positions included were 1st+2nd+3rd+Noncoding. All ambiguous positions were removed for each sequence pair. There were a total of 657 positions in the final dataset. Evolutionary distance analyses were conducted in MEGA6 (Tamura et al. 2013). Two frequency distribution diagrams of all pair-wise intra- and inter-specific distances were produced to further evaluate species divergence in *Cryptops*. All samples of each species were grouped in the first analysis, while *Cryptops parisi* was split into the three separate clades *Cryptops parisi* sensu stricto, *Cryptops parisi sebini* and *Cryptops parisi* lineage3 in the second analysis.

## Results

### Phylogenetic analysis

**Figure 2.**
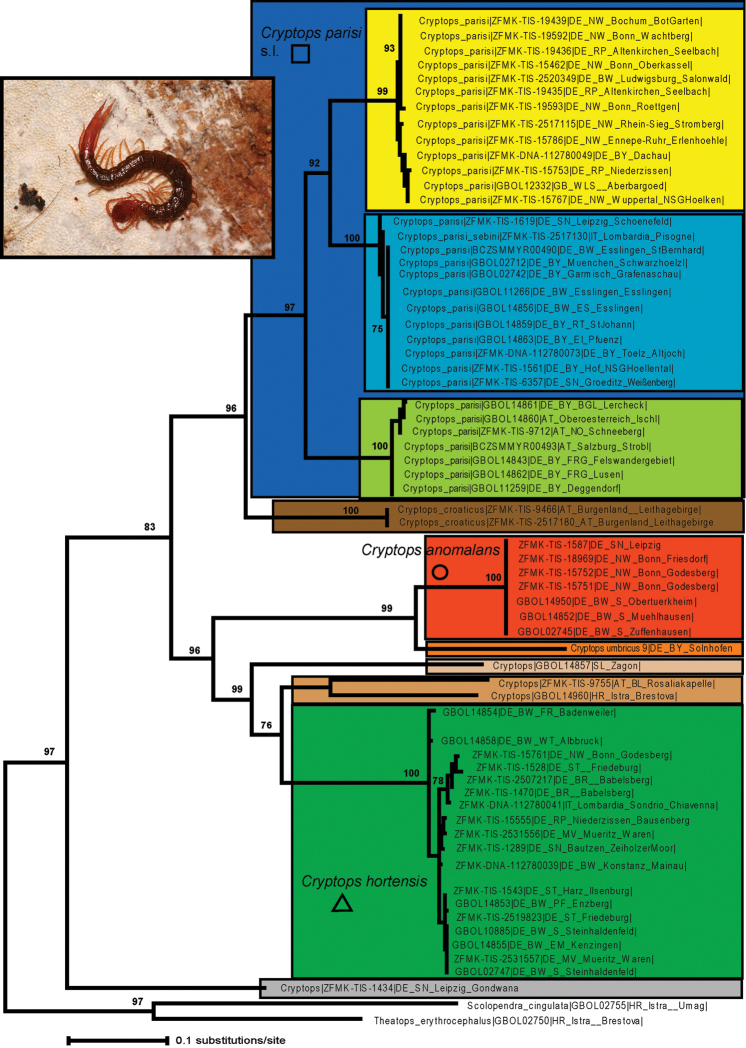
Maximum likelihood tree under the GTR+G+I model, 1000 bootstrap replicates. Colours and symbols correspond to Maps (Figs 1, 4). Country of origin given after specimen number: **AT** = Austria; **DE** = Germany; **GB** = Wales; **HR** = Croatia; **IT** = Italy; **SL** = Slovenia. Photograph shows a specimen of *Cryptops parisi* s.s. from Breckerfeld (photo A. Steiner), western Germany. For full data on all specimens, see Table 1.

The monophyly of the genus *Cryptops* is strongly supported (97%) in our tree (Fig. 2). One undetermined *Cryptops* sp. collected from the tropical rainforest greenhouse at the Leipzig Zoo in eastern Germany (Fig. 1: 55) is in a basal position juxtaposed to all other *Cryptops* specimens (Fig. 2). The remaining Central European *Cryptops* are split into two clades, of which only the *Cryptops parisi*/*Cryptops croaticus* clade receives high statistical support (96%). The unsupported clade unites *Cryptops umbricus* and *Cryptops anomalans*, three specimens of uncertain identity, and *Cryptops hortensis* (Fig. 2). *Cryptops anomalans* is in a basal position regarding this second (unsupported) clade with a single haplotype spread all over Germany, forming a monophylum with *Cryptops umbricus* from Solnhofen, Germany, representing the first record from this country (Fig. 1: 46). The uncertain *Cryptops* sp. from Slovenia is a sister group to a weakly supported clade (76% bootstrap support) uniting two unidentified *Cryptops* sp. specimens with *Cryptops hortensis* (Fig. 2). The latter two unidentified *Cryptops* sp. specimens from eastern Austria and Croatia are grouped together, but this grouping is not statistically supported.

**Figure 3.**
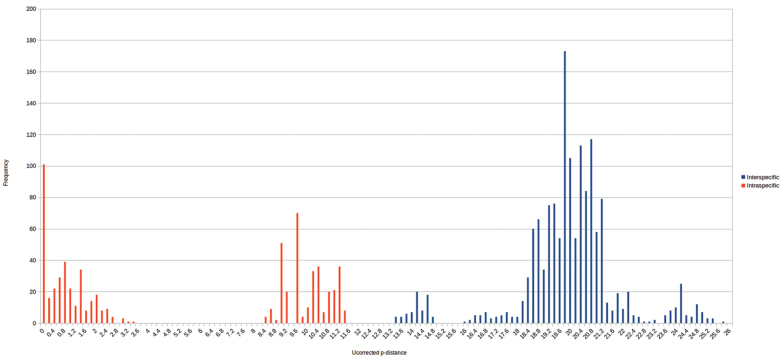
Frequency distribution of pairwise intraspecific (blue) and interspecific (red) distances. All lineages of *Cryptops parisi* treated as one species, *Cryptops parisi* sensu lato. Basic table see Suppl. material 1.

**Figure 4.**
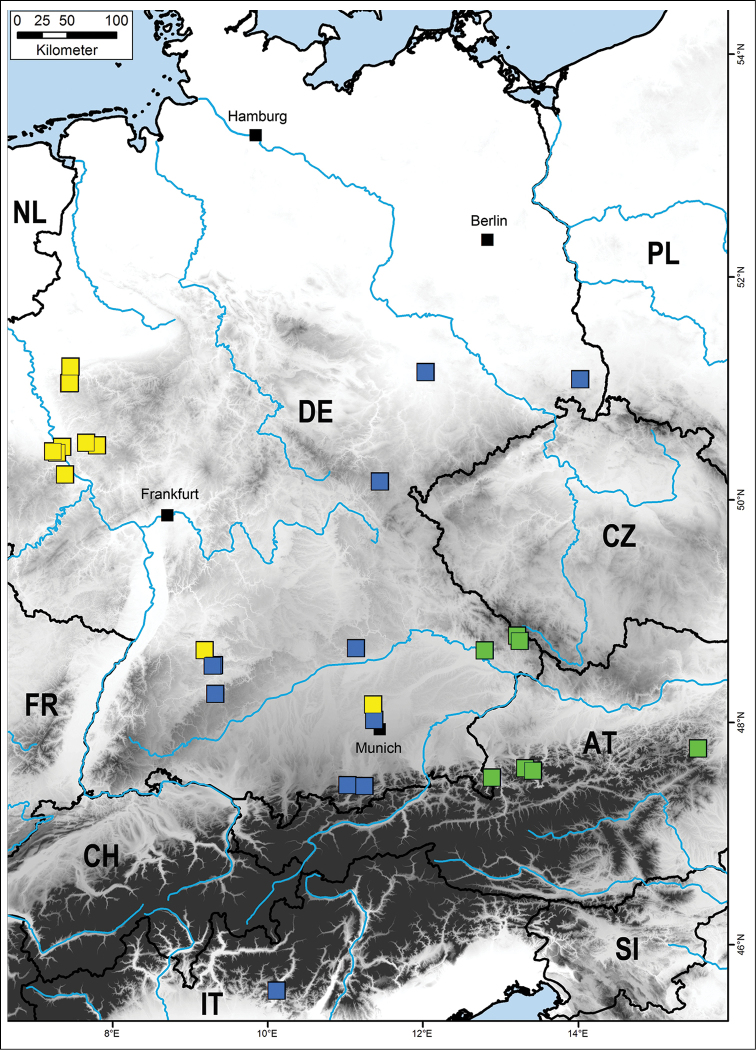
Distribution map of all successfully sequenced Central European specimens of *Cryptops parisi*. Different colours mark the three different clades. Yellow = *Cryptops parisi* sensu stricto; blue = Cryptops parisi sebini; green = *Cryptops parisi* lineage 3 (potentially Cryptops cf. hortensis sensu Pichler 1987).

The monophyly of the 18 specimens of *Cryptops hortensis* is strongly supported (100%). Of the shallow clades inside *Cryptops hortensis* (Fig. 2), only one, a clade uniting five different haplotypes from Italy, eastern and western Germany (Fig. 4), receives some statistical support (78%). Interestingly, a second specimen from Friedeburg, Saxony-Anhalt, the same locality as one of the five haplotypes mentioned above (see Table 1), groups within a separate clade (Fig. 2).

The clade uniting *Cryptops parisi* sensu lato and *Cryptops croaticus* receives high statistical support (96%). While both specimens of *Cryptops croaticus* show the same haplotype, the 32 specimens of *Cryptops parisi* s. l. are separated into three statistically well-supported (99–100%) clades. The basalmost clade (Fig. 2) includes seven specimens and represents three different haplotypes from the eastern alpine region (Fig. 4: green). The remaining two clades of *Cryptops parisi* are clearly related (92% support); one represents a western clade (Fig. 4: yellow) and the other is found slightly more to the east (Fig. 4: blue) and also includes the topotypoid of the subspecies *Cryptops parisi sebini*.

### Distance analysis

**Figure 5.**
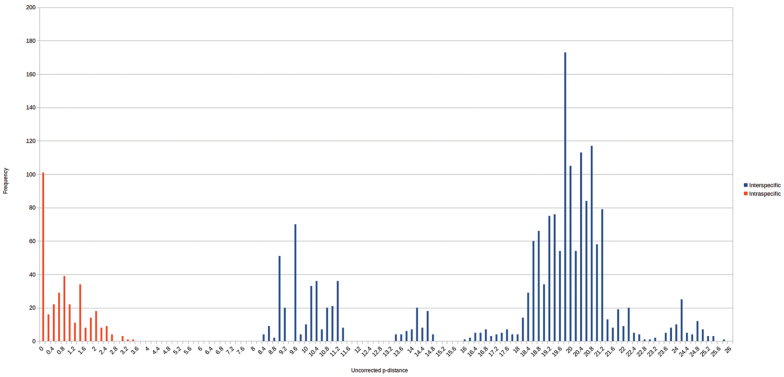
Frequency distribution of pairwise intraspecific (blue) and interspecific (red) distances. The three lineages of *Cryptops parisi* treated as different species. Basic table see Suppl. material 1.

*Cryptops* specimens differ from the outgroups *Scolopendra* and *Theatops* by 19.8–25.7% (Supplementary Material 2). Interspecific and intraspecific distances of the different nominal *Cryptops* species show no overlap (Fig. 3). Interspecific distances lie between 13.4–21.1% (Fig. 3), with the lowest observed between *Cryptops croaticus* and *Cryptops parisi* s. l. (13.4–14.8%) as well as between *Cryptops anomalans* and *Cryptops umbricus* (13.9%). Otherwise, interspecific distances are always >16%, with the highest value of >20% observed between *Cryptops anomalans* and *Cryptops hortensis*, as well as between *Cryptops parisi sebini* and *Cryptops hortensis*. Intraspecific distances are between 0–11.3%. However, intraspecific distances are low, 0–3.3%, if we treat the three distinct lineages of *Cryptops parisi* as distinct species (Fig. 5).

## Discussion

### Distance analysis

Clear intraspecific distances in German or even Central European *Cryptops* are low. The specimens filling the majority of our barcoding gap between 3 and 11.3% are the different lineages of *Cryptops parisi*, which differ by 8.4–11.3% from one another (Fig. 3) and might represent distinguishable taxonomic units (see below). Two specimens directly at the edge between inter- and intraspecific distances (Fig. 5), the two *Cryptops* “sp. 2” specimens from Austria and Croatia (15.9%), require a careful re-study (see below).

### The biogeographic and ecological pattern of *Cryptops hortensis* and *Cryptops parisi* in Central Europe

*Cryptops parisi* and *Cryptops hortensis* belong to the South European and Central Asiatic European chorotypes respectively (Zapparoli 2006). In central Europe *Cryptops hortensis* and *Cryptops parisi* s. l. seem to exclude each other either geographically or ecologically. In the lowland areas of north-western Germany and in the Upper Rhine valley it is usually *Cryptops hortensis* that occurs, while in the lower mountain ranges usually *Cryptops parisi* is present. Nevertheless, *Cryptops parisi* mainly avoids higher altitudes. In the eastern part of Germany *Cryptops parisi* dominates.

*Cryptops parisi* is generally classified as a mesophilous woodland species (Spelda 1999, Minelli and Iovane 1987, Voigtländer et al. 1997), but may also occur outside of forests, especially in northern Germany where more anthropogenic influenced places are inhabited.

The two clearly differentiated genetical lineages in *Cryptops parisi* s. s. in Germany (see below) are reflected in distinct ecological differences in the preferred habitats between the western and eastern parts of Germany. In the more Atlantic areas in the West, the species prefers woodland like in its main distribution area. In the more continental influenced East, *Cryptops parisi* inhabits open-dry habitats such as dry meadows, mesoxeric meadows and their successional shrub-stages, as well as dwarf-shrub heaths (Voigtländer 2003a, 2003b, 2005).

### A single haplotype in German *Cryptops anomalans*

*Cryptops anomalans* is viewed as a species introduced to Germany and England (Eason 1964; Voigtländer 1988). Specimen records are rare, e.g. the species has only recently been recorded from Germany, where it only occurs in localized areas, usually in parks or gardens (Lindner 2010, Decker and Hannig 2011). Our findings show that a single haplotype (Fig. 2) is present in western, eastern and southern Germany (Fig. 1), while all other *Cryptops* (see Fig. 3), as well as Geophilomorpha species (Wesener et al. 2015) show different haplotypes across a large geographical area. An identical haplotype from different localities might be interpreted as recent human introductions from a homogenous source population or a rapid spread of *Cryptops anomalans* in Germany.

### First record of *Cryptops umbricus* in Germany

Our analyses first showed one outlier *Cryptops anomalans* specimen from Solnhofen, Bavaria (Fig. 1), which strongly differs by 13.9% from the common German haplotype. This was the only specimen of *Cryptops anomalans* in a previous analysis involving German centipedes (Spelda et al. 2011). A morphological check against similar species showed that it was indeed not *Cryptops anomalans* but represents *Cryptops umbricus*, a first record for Germany. This finding shows the usefulness of the barcoding method in detecting previously unrecorded species.

### At least three undetermined *Cryptops* species in Central Europe

*Cryptops* sp. 1 is only represented in our dataset by a single specimen from Slovenia, which is unfortunately missing the pre-ultimate legs and can therefore not easily be determined morphologically.

*Cryptops* sp. 2 is represented by two specimens that are separated by a wide genetic distance of 15.9%. This distance usually falls right into the lower limit observed between different *Cryptops* species (Fig. 3). The two specimens are from the eastern lowlands of Austria (Burgenland) and Croatia (Brestova). Unfortunately, the Austrian specimen is heavily damaged with missing posterior segments, which prevents any determination. As both specimens of *Cryptops* sp. 2 are related, but potentially not conspecific, they are discussed here together.

These two specimens are similar to *Cryptops hortensis*, but are missing the ventral furrow on the prefemora of the ultimate leg pair. An available name for one of these lines might be *Cryptops rucneri* Matic, 1966. This species was synonymised with *Cryptops hortensis* by Koren (1986), followed by Spelda (1999), but treated as a valid species later (Stoev (2002). The presently discovered genetic diversity brings this name into consideration again. One argument for the identity of one of our lines with *Cryptops rucneri* is the configuration of the prefemur of the ultimate legpair, where Matic (1966) did not mention a ventral furrow. Although Matic (1966, 1972) did not describe and depict the poison gland in great detail, his figures clearly show that in both *Cryptops hortensis* sensu Matic (1972a) and *Cryptops rucneri*, the calyx of the poison glands lie mainly in the femur and tibia of the forcipule. Matic also records *Cryptops rucneri* from Italy (Matic 1967), Austria: Carynthia (Matic 1972b), and Slovenia (Matic 1979).

Maybe this specimen is the same species to which Pichler (1987) refers to as Cryptops cf. hortensis from North Tyrol. The shape of the poison gland was not illustrated for Cryptops cf. hortensis. The poison gland allows a clear separation from *Cryptops parisi* even in very early stages. Without checking the poison gland, juvenile specimens of *Cryptops parisi*, which lack the characteristics of adult specimens (a central depression on the forcipular tergite and the pair of occipital sutures), can be easily mistaken for *Cryptops hortensis*. Pichler (1987) records an unidentate labrum for Cryptops cf. hortensis, as does Matic (1966) for *Cryptops rucneri*. Pichler’s (1987) fig. 18 of the 21st pleurocoxa corresponds to fig. 4 of Matic (1966) for *Cryptops rucneri*.

Of the two specimens of *Cryptops* sp. 2, the one from Brestova is the most probable to represent *Cryptops rucneri*. This specimen was collected only 30 kilometres distant from the type locality of *Cryptops rucneri* and shows the characteristic elongated 20th leg pair, which is unfortunately missing in the other specimen (as well as in our *Cryptops* sp. 1). Nevertheless, while having only three sequences of these eastern *Cryptops hortensis*-relatives and without being able to provide a revision of the *hortensis*/*rucneri*-complex we prefer at the moment to keep these specimens under the name *Cryptops* sp.

*Cryptops* sp. 3, previously determined as Cryptops cf. doriae Pocock, 1891 is only known from the Leipzig Zoo in eastern Germany, where it was collected in a large tropical greenhouse (Decker et al. 2014). It was provisionally identified as *Cryptops doriae*, a member of the *doriae*-group, which is characterized by having teeth on femur, tibia and tarsus of the ultimate legs (Lewis 2011). *Cryptops doriae* was already reported from a tropical biome in England (Lewis 2007) and is so far the only introduced tropical *Cryptops* species with records in Europe (Stoev et al. 2010). A BLAST search of our specimen against the sequences of *Cryptops doriae* already deposited on GenBank (11.2015) reveals a large genetic distance between our specimen and the ones from the Pacific, which is the reason we refer to our specimen as *Cryptops* sp. 3.

### First record of *Cryptops croaticus* in Austria

*Cryptops croaticus* was originally described from Bakar (formerly Buccari) in Croatia (Verhoeff 1931) and subsequently recorded from other localities in Croatia, Slovenia and Bosnia-Herzegovina (Matic 1966, 1979, Kos 1992), Greece (Matic 1976), Bulgaria (Stoev 1997a, 2002), and Italy (Matic 1960, 1968, Matic and Darabantu 1971, Minelli 1985, 1992). Currently, *Cryptops croaticus* seems to be absent or not yet found in Hungary (Dányi 2008). One subspecies (*Cryptops croaticus burzenlandicus*) was described from Romania (Verhoeff 1931) and was subsequently synonymised with the nominal subspecies (Matic 1972a), another subspecies, *Cryptops croaticus albanicus*, has been described from Albania (Verhoeff 1934) and was later synonymized under *Cryptops anomalans* (Stoev 1997b). Several subspecies have been described from Italy, namely *Cryptops croaticus bergomatius* (Verhoeff 1934), *Cryptops croaticus longobardius* and *Cryptops croaticus baldensis* (Manfredi 1948), subsequently cited by Conci (1951) and Boldori (1969). Based on this wide distribution, the occurrence of *Cryptops croaticus* in Austria is not unexpected. In Austria, it is currently only known from a southern exposed slope, which is home to numerous relic species adapted to a warmer climate. *Cryptops croaticus* shares its habitat with the recently rediscovered population of *Scolopendra cingulata* in Austria (Oeyen et al. 2014), as well as the thermophilic beetle *Carabus hungaricus* and other thermophilic animals (Böhme et al. 2014). However, the determination of our specimens as *Cryptops croaticus* is only based on the characters given in the original description (Verhoeff 1931) as no better description exists. Numerous important characters, such as the last leg pairs, are unfortunately missing in our specimens. A revision of *Cryptops croaticus* is urgently needed (Matic 1966) as it may be that some of the nominal subspecies represent independent species. One way to clarify this is to collect and sequence topotypic material. Once *Cryptops croaticus* has been properly revised, a re-evaluation of the Austrian specimens should be undertaken.

### The three lineages of *Cryptops parisi* sensu lato

The three lineages of specimens placed in *Cryptops parisi* by morphological characters differ 8.4–11.3% from one another, while their intra-lineage genetic distance is much lower at 0–1.1%. A large barcoding gap becomes clearly visible in our dataset when we treat the three different lineages of *Cryptops parisi* as separate species (Figs 4, 5). Endosymbionts like *Wolbachia* (Hurst and Jiggins 2005) are an unlikely explanation for the different lineages, as such endosymbionts have never been recorded in the Myriapoda (Witzel et al. 2003).

One lineage clearly represents the *Cryptops parisi* sensu stricto (Fig. 2: yellow). This group shows a western distribution in Germany, with a single specimen from southern Germany (Fig. 4). The type locality of *Cryptops parisi* is, as the species epithet implies, Paris, France. Our only sample from Great Britain (Wales) also falls into this group. Intra-lineage variation is low with 0–1.7%. Inner structure of the lineage is limited due to the small genetic distances inside the group, but one group containing only few haplotypes differing in a single or two basepairs from one another is well-supported. This group contains specimens from western Germany, as well as a single specimen each from southwestern (ZFMK-TIS 2520349) and southeastern Germany (ZFMK-DNA-112780049), but these two were collected in a park and a garden.

A second distinct group (Fig. 2: Blue) contains the topotypic specimen of the subspecies *Cryptops parisi sebini* Verhoeff, 1934. *Cryptops parisi sebini* was recently synonymised under *Cryptops parisi* because no morphological differences could be detected (Lewis 2011). However, the distinctiveness of the subspecies *Cryptops parisi sebini* should be re-evaluated, as our genetic data supports this monophyletic subspecies (100% bootstrap support) with a high genetic distance to *Cryptops parisi* s. s. (8.4–9.4%) in combination with low intra-lineage variation (0–0.6%) despite the large geographical distances between the analyzed specimens from Italy and eastern Germany. This *Cryptops parisi* group 2 shows a distribution to the east of *Cryptops parisi* s. s., with localities in eastern northern Italy and the eastern half as well as the south of Germany (Fig. 4). Another name potentially available for this clade is *Cryptops parisi rhenanus* Verhoeff, 1931, which is characterized by its extremely elongated calyx of the poison gland (Verhoeff 1931). If both names turn out to represent the same species, this taxon would have priority over *Cryptops parisi sebini*, with which it is compared in the original description (Verhoeff 1934). Unfortunately, Verhoeff (1931) never designated a type for *Cryptops parisi rhenanus*. The specimens represented in the Bavarian State Collection of Zoology originate from a large number of localities.

The specimens of *Cryptops parisi* s. l. belonging to a third group (Fig. 2: green), referred here as *Cryptops parisi* lineage 3, are morphologically and genetically distinct and may also be identical to the specimens of Cryptops cf. hortensis in the literature (Pichler 1987, Lewis 2011). Our specimens of *Cryptops parisi* lineage 3 come mainly from alpine habitats in Austria and Germany. In the most recent revision of the species group (Lewis 2011), these specimens were listed in the key under *Cryptops parisi*, but with remarks concerning its unique morphology. Coxal pores are too numerous (~50) for *Cryptops hortensis* and more closely resemble the lower end of *Cryptops parisi*. Other morphological characters prompted Lewis (2011) to place these specimens in his key under *Cryptops parisi*, an affinity confirmed here by our genetic analysis.

However, the large genetic distance of 10–11.3% between *Cryptops parisi* lineage 3 and *Cryptops parisi* s. s. as well as to the lineage containing *Cryptops parisi sebini*, combined with a low intraspecific distance (0–1.1%) are clear indications that these specimens might represent a species of its own.

To find names for our two eastern lines of *Cryptops parisi* one has to go back to C. L. Koch, who described three *Cryptops* species from around Regensburg, Germany: *Cryptops ochraceus* C. L. Koch, 1844 from the Keilstein (a calcareous mountain east of Regensburg), *Cryptops sylvaticus* C. L. Koch, 1844 from the Naab-valley (north of Regensburg) and *Cryptops pallens* C. L. Koch, 1847 from the moat of Regensburg. More information on these species, such as the precise type localities and more detailed descriptions, are provided in Koch (1863), which has often resulted in these species erroneously being assigned to the date of this second publication.

Attems (1930) indicated that it would be impossible to assign these species to either *Cryptops hortensis* or *Cryptops parisi*, while Matic (1972a) simply synonymized them with *Cryptops hortensis*. Both did not take note of the central depression, often darker than the adjacent parts of the tergite, as a character separating *Cryptops parisi* from *Cryptops hortensis*, at least for adult specimens from southern Germany (own observation, JS). This depression is also described by Attems (1930) as existing in some *Cryptops parisi* specimens, but is not otherwise mentioned in the available keys separating the two species (Attems 1930, Brölemann 1930, Verhoeff 1931, Eason 1964, Matic 1972a, Koren 1982, 1986, Iorio and Geoffroy 2008). Verhoeff (1934) also described this character in *Cryptops parisi sebini*. Koch (1863) clearly states and depicts the depression for his species *Cryptops sylvaticus* and *Cryptops ochraceus*. It seems only to be missing in *Cryptops pallens*, which represents a juvenile specimen. Another argument against a synonymy of these species with *Cryptops hortensis* is the absence of the latter species in our extensive collections from eastern Bavaria. Topotypoids of *Cryptops ochraceus* have already been collected and might clarify this species in the near future.

It should be noted that Matic (1972a) depicts a *Cryptops parisi* with a short poison gland. This specimen surely represents a different species.

### Outlook/future studies

Future prospects should include the parallel sequencing of nuclear genes to confirm the relationships drawn from the mitochondrial barcoding fragment. To clarify the taxonomic relationships within *Cryptops parisi*, it would be important to collect further samples to enable an extensive morphological evaluation.

## Supplementary materials

Supplementary material 1 Full specimen information Data type: occurence Explanation note: Full specimen information. This dataset is made available under the Open Database License (http://opendatacommons.org/licenses/odbl/1.0/). The Open Database License (ODbL) is a license agreement intended to allow users to freely share, modify, and use this Dataset while maintaining this same freedom for others, provided that the original source and author(s) are credited.homas Wesener, Karin Voigtländer, Peter Decker, Jan Philip Oeyen, Jörg Spelda

Supplementary material 2 Uncorrected P-Distance Data type: measurements Explanation note: Uncorrected P-Distance between the 66 analyzed specimens. This dataset is made available under the Open Database License (http://opendatacommons.org/licenses/odbl/1.0/). The Open Database License (ODbL) is a license agreement intended to allow users to freely share, modify, and use this Dataset while maintaining this same freedom for others, provided that the original source and author(s) are credited.homas Wesener, Karin Voigtländer, Peter Decker, Jan Philip Oeyen, Jörg Spelda
